# Energy-use efficiency of organic and conventional plant production systems in Germany

**DOI:** 10.1038/s41598-024-51768-3

**Published:** 2024-01-20

**Authors:** Lucie Chmelíková, Harald Schmid, Sandra Anke, Kurt-Jürgen Hülsbergen

**Affiliations:** Chair of Organic Agriculture and Agronomy, TUM School of Life Sciences Weihenstephan, Technical University of Munich, Liesel-Beckmann-Str. 2, 85354 Freising, Germany

**Keywords:** Plant sciences, Environmental impact, Fossil fuels

## Abstract

Sustainable and efficient energy use in agriculture helps tackle climate change by reducing fossil energy use. We evaluated German farming systems by analysing energy input and output. Data from 30 organic and 30 conventional farms (12 arable, 18 dairy farms each) between 2009 and 2011 was used. Energy input, output, and the influence of farm type, farm structure, and management intensity on energy-use efficiency (EUE) were analysed for crop production using the farm management system REPRO. Conventional farms (CF) always had higher energy input. The energy input for organic farms (OF) was 7.2 GJ ha^−1^ and for CF 14.0 GJ ha^−1^. The energy output of CF was also higher. Reductions were higher in energy input than in energy output. In 73.3% of the farm pairs, OF were more energy efficient than CF. The EUE was comparable with CF on 10% of OF and for 16.7% of CF the EUE was higher suggesting better fossil energy utilization. EUE can be increased when reducing fossil energy inputs through more efficient machinery, reduction of agrochemicals, precision farming, the use of renewable energy or energy retention, and by increasing yields. A reduction of inputs is urgently required to lower the (political) dependence on fossil energy.

## Introduction

The intensification of agriculture, accompanied by the increased use of mineral fertilizers and pesticides and mechanization, has led to the increasing use of fossil energy over the decades^1–4^. In modern agriculture today, in addition to the direct use of fossil energy in production processes, an extremely high fossil energy input is required for the production of capital goods and operating resources, especially for the production of mineral nitrogen fertilizer^5,6^, which accounts for more than 50% of the total energy input^7^. Agricultural production still relies heavily on the consumption of fossil energy in almost every production step. However, there are many approaches to saving fossil energy, e.g. through the use of more efficient and innovative machinery^8^, reduced use of agrochemicals^9^, precision farming^10^, and the use of renewable energy^11^.

Current technologies and the management of agricultural systems offer opportunities to improve energy-use efficiency, reduce the dependency on non-renewable energy sources, and mitigate greenhouse gas emissions^12,13^. The extremely high prices for mineral fertilizer in recent months show the dependency on fossil fuels coupled with gas prices. Agricultural systems based on the use of fossil energy increasingly appear to be very vulnerable systems that are not sustainable and contribute to climate change. Therefore, there is an increasing conflict with the German/EU reduction targets for energy consumption and GHG emissions.

One way of drastically reducing the energy input in crop production is to forego the use of mineral fertilizer and chemical plant protection products—a basic principle of organic farming. However, this could have many implications, such as higher energy input (more mechanical procedures, tillage, and weed control) and yield reduction (energy output), and therefore a negative impact on energy efficiency. Therefore, it is not clear whether organic farming is more energy efficient^14–18^. The development of more energy- and resource-efficient organic and conventional farming (CF) systems is of great importance for the future. Efficient energy use is one of the most important conditions for sustainable agriculture^19^. Generally, organic farming (OF) is thought of as being environmentally sustainable and energy-efficient due to the absence of chemical pesticides and mineral nitrogen fertilizers. As Pimentel et al.^20^ suggested, the interdependence of energy economics and the environment, energy efficiency and the transition to renewable energy sources is critical.

There are systemic differences between OF and CF. Mineral nitrogen fertilizers and chemical pesticide inputs are used in CF, whereas biological plant protection and symbiotic N_2_ fixation and organic manure are used in OF. The use of biologically-fixed nitrogen represents considerable energy saving compared to the energy-intensive Haber–Bosch process^21^. According to many studies, OF is characterised by more intensive tillage, cover crop establishment^22^, manure application^23^, and a greater number of field operations required for mechanical weed control^21,24,25^. On the other hand, there are very extensive agricultural systems in OF with a low number of management processes. In addition, mechanical weed control requires less energy than the production of plant protection products. Organic fertilizers are also used in CF, however the stocking density is higher than in OF, and therefore more organic fertilizer (e.g. slurry) has to be applied.

The main components of an on-farm energy balance calculation are energy input, energy output, and energy-use efficiency (EUE). These parameters characterize agricultural systems and assess the systems from a sustainability perspective. Almost all indicator-based sustainability management systems in agriculture contain energetic indicators, such as the input of fossil energy or energy efficiency. In the EU, energy use is one of the 28 agri-environmental indicators of the Common Agricultural Policy (CAP). Further, energy consumption is an indicator used in the United Nations Sustainable Development Goals^26^. In Germany, energy balancing is an indicator in the DLG (German Agricultural Society) Sustainability Standard for farms.

Studies comparing organic and conventional farming systems have generally reported significant energy savings in OF. Generally, fertilization and mechanization are the two main components of energy input in crop production systems^27^. OF is well known for its low energy input^28,29^. Total energy input was 37–50% lower in OF than in CF^30–32^. About one third of the total energy input in crop production systems, depending on the crop^30,33^, corresponds to the production of fertilizers^34^. Low fossil energy input is often, but not always, associated with low energy output. Crops do not always use the applied nitrogen fertilizers and manures efficiently^35^. OF tries to achieve high nitrogen efficiency. The yield gap between CF and OF can be reduced by using crop rotation effects, biological N_2_ fixation and biological crop protection^21^. The lower yield and energy output of OF (yield gap) are often discussed when comparing the two systems. Globally, organic yields were 75% of conventional yields^36^ and required more land to produce the same amount of products^32^. Nevertheless, yield differences varied greatly over the years, from 45 to 90%, and depended on crop type^37^. For cereals, Pimentel et al.^38^ recorded less fossil energy input and higher yields in OF. Helander and Delin^39^ reported very low yield levels in OF compared to CF and consequently lower EUE than in CF. Nevertheless, according to many authors^19,23,27^, higher conventional yields were not high enough to compensate for the additional energy consumption, indicating that OF was more efficient. The EUE will decrease or increase depending on whether the yield depression is proportionately greater than the reduced energy input, or vice versa^21^.

There are already many studies on the EUE of OF and CF. As suggested by Baum et al.^40^, many studies are based on questionnaires and aggregate statistics from surveys. Direct comparisons of the systems are difficult. Many energy balances, even in OF and CF system comparisons, are incomplete and do not take into account the whole system, i.e. all relevant energy flows, inputs, and outputs. For example, it is difficult to quantify the cumulative energy input for machinery manufacture and therefore this is often simply not included. The contradictory results in the comparisons of OF and CF are caused by (a) not enough attention to the variability of the cultivation systems within organic and conventional farming. Often, two systems are compared without considering whether they are representative or not (in terms of crop rotation, fertilization intensity, yield level, etc.). (b) There are enormous differences in performance (yields) and management intensity (fertilization) depending on farm structure. (c) Site conditions and their yield potential are not taken into account. Energy efficiency is dependent on soil and climatic conditions. On-farm research that takes the complete farming system into account is needed. Nevertheless, it is important to analyze subsystems, e.g. plant production as a system, to determine how energy-efficiently plant biomass can be produced. This would enable farming systems to be optimized.

In 2009, a network of pilot farms (www.pilotbetriebe.de) was established in Germany to analyse and compare the climate impacts and energy and nutrient efficiency of organic and conventional farming systems. Extensive data were collected from the pilot farms in regions with different soil, climate, and management conditions and analysed using models. In three study regions, energy balances were calculated for 10 farm pairs consisting of adjacent organic and conventional farms. The very comprehensive, detailed data set from the network was used to evaluate the specific energy flows in organic and conventional farming. The study aims to analyse and compare the energy balance of organic and conventional plant production systems. In these plant production systems, cash crops and fodder are produced from arable land and permanent grassland. Organic and conventional plant production systems are represented by two farming types—arable and dairy farming systems. The energy balances are used to determine the following energetic indicators (a) energy input, (b) energy output and (c) energy-use efficiency (EUE). Further, the relationship between energy input and energy output was analysed.

The energy balances are intended to analyse (a) system-related differences in the energy balance and energy-use efficiency of organic and conventional plant production systems, (b) the variability of energetic indicators within organic and conventional plant production systems, (c) the influence of increasing fossil energy inputs on energy output and energy-use efficiency. The results are used to draw generalised conclusions for the further energetic optimisation of organic and conventional systems.

## Results

The results show great variability in farm management (Table 1). The farms differed in the amount of livestock on the farm, the maximum for CF was 2.72 LU ha^-1^a^-1^, and in OF the maximum was 1.56 LU ha^-1^a^-1^ (livestock units (LU) are a standardized measure for comparing livestock density with one LU being equal to a cow weighing about 500 kg). Hence, the proportion of grass-clover in OF (DF: 40%, AF: 19%) differed greatly among the farms as well. Grain legumes amounted to 4% of the crop rotation in DF and 11% in AF in organic farming. On the other hand, in CF there were even some farms without any grass-clover. Maize for fodder production and root crops made up a large proportion of the conventional crop rotations. In organic DF, cultivated grass-clover and winter wheat were predominant. More details on differences in the forage crops of the pilot farms are described in Frank et al. ^41^. Furthermore, there were differences in crop diversity and composition of mixtures undersown in cereals (e.g. species-rich grass-clover mixtures) leading to, for example, differences in ground cover. Farm characteristics affected the energy balance.

**Table 1:** Characteristics of the pilot farms^45^.

Parameter	Unit	Organic farming system		Conventional farming system	
		Arable farm	Dairy farm	Arable farm	Dairy farm
Number of farms		12	18	12	18
Elevation	m a.s.l	204 (0–588)	260 (3–780)	213 (0–588)	257 (1–780)
Precipitation	mm a^−1^	771 (591–1109)	863 (536–1507)	771 (591–1109)	863 (536–1507)
Average temperature	°C	8.5 (7.5–9.7)	8.5 (6.9–10.8)	8.5 (7.5–9.7)	8.5 (6.9–10.8)
Soil quality^a^		56 (41–75)	43 (23–64)	58 (40–78)	47 (25–68)
Area	ha	195 (57–511)	183 (30–1317)	261 (65–1224)	104 (30–312)
Cropland	%	94 (73–100)	54 (0–96)	97 (81–100)	60 (0–85)
Cereals	% crop land	57 (36–76)	41 (0–68)	64 (44–95)	40 (0–69)
Grain legumes	% crop land	11 (0–17)	4 (0–16)	0	1 (0–8)
Root crops, maize	% crop land	8 (0–32)	8 (0–24)	14 (0–47)	34 (11–70)
Grass-clover	% crop land	19 (6–33)	40 (17–81)	3 (0–17)	11 (0–46)
Yield of winter wheat	t ha^−1^ a^−1^	3.9 (3.0–5.2)	3.8 (2.9–5.1)	8.6 (7.1–10.0)	7.7 (5.3–9.7)
Undersowing	% crop land	8 (0–24)	9 (0–28)	0	3 (0–59)
Catch crops	% crop land	18 (0–38)	12 (0–31)	11 (0–42)	13 (0–59)
Crop diversity^b^		2.36 (1.78–3.04)	2.05 (1.01–2.95)	1.62 (0.91–-2.41)	1.54 (0.84–-2.04)
Livestock^c^	LU ha^−1^ a^−1^	0.01 (0–0.08)	0.87 (0.27–1.56)	0	1.46 (0.54–-2.72)

Units in parentheses represent minimum and maximum values, respectively. ^a^Müncheberger Soil Quality Rating (0–100 points) for assessing the agricultural yield potential of German soils^68^, ^b^Crop diversity is calculated as the proportion of the individual crop of the total cropped area, ^c^ Livestock unit (LU) unit is a reference unit which facilitates the aggregation of livestock from various species and defined age classes, via the use of specific coefficients.

### Energy input

**Figure 1:**
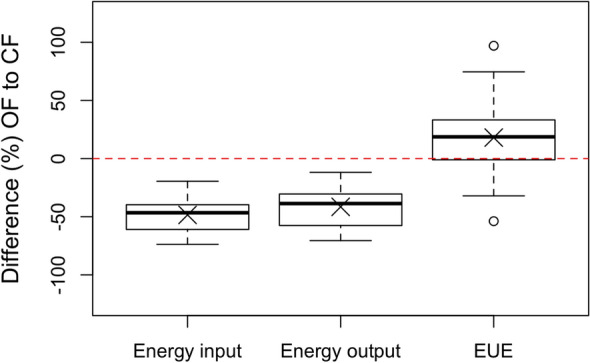
Comparison (%) of energy input, energy output and energy-use efficiency (EUE) between organic and conventional farming. Thick line within the box plot represents the median, “x “ represents the mean value.

Generally, energy input was lower in OF in all comparison pairs (Fig. 1). Considering all farm systems, the yearly average total energy input, including direct and indirect inputs (seed, plant protection products, mineral and organic fertilizer, machinery), was higher in CF (14.0 GJ ha^−1^) than in OF (7.2 GJ ha^−1^) in all comparison pairs (Table 2). Energy input in OF ranged from 4.0 to 10.7 GJ ha^−1^, and in CF from 10.0 to 17.1 GJ ha^−1^. The total input, when organic fertilizers were not taken into account, ranged from 2.0 to 8.4 GJ ha^−1^ in OF, and in CF from 4.6 to 14.9 GJ ha^−1^ (Table 2).

**Table 2:** Energy input, energy output and energy-use efficiency (EUE) of organic (OF) and conventional plant production (CF) systems on 60 farms in Germany.

	Energy input (GJ ha^−1^ a^−1^)		Energy input without organic fertilizers (GJ ha^−1^ a^−1^)		Energy output (GJ ha^−1^ a^−1^)		EUE		EUE without organic fertilizers	
	OF	CF	OF	CF	OF	CF	OF	CF	OF	CF
Mean	7.2	14.0	4.6	10.3	101.0	171.8	14.4	12.3	25.2	18.7
Median	6.7	13.7	4.1	10.8	100.3	174.8	14.7	12.6	22.5	15.9
Min	4.0	10.0	2.0	4.6	51.6	109.7	6.8	8.4	8.4	8.7
Max	10.7	17.1	8.4	14.9	165.0	226.3	26.2	15.6	61.5	42.6

The highest total energy input (11.3–17.1 GJ ha^−1^, Table 3) was recorded on conventional dairy farms, followed by conventional arable farms (10.0–15.1 GJ ha^−1^). The energy input in OF was significantly lower for both types of organic farming. The energy input on arable farms was 4.0–10.7 GJ ha^−1^, on dairy farms 4.9–10.2 GJ ha^−1^. In arable OF, the highest energy inputs were the direct energy inputs (3.8 GJ ha^−1^) due to high tillage intensity (ploughing) and intensive mechanical weed control. Diesel use was lower on organic dairy farms due to the high proportion of grassland and pasture (energy-extensive systems). The lowest direct input was observed in dairy farms in OF, it was lower than on arable farms (OF) and dairy farms in CF.

**Table 3:** Energy input, energy output and EUE in organic and conventional plant production systems, mean values for the years 2009–2011.

Parameter	Unit	Organic plant production system		Conventional plant production system		Mean	*p*
		Cash crop production (Arable farming) (n = 12)	Forage and grassland production (Dairy farming) (n = 18)	Cash crop production (Arable farming) (n = 12)	Forage and grassland production Dairy farm (n = 18)		
Total energy input	GJ ha^−1^ a^−1^	6.8^a^	7.4^a^	13.4^b^	14.4^b^	10.6	***
		(4.0–10.7)	(4.9–10.2)	(10.0–15.1)	(11.3–17.1)		
Energy input without organic fertilizer	GJ ha^−1^ a^−1^	5.7^b^	3.8^a^	12,5^d^	8.9^c^	7.4	***
		(3.8–8.4)	(2.0–5.8)	(9.6–14.9)	(4.6–14.4)		
Direct energy input (diesel)	GJ ha^−1^ a^−1^	3.8^a^	2.7^b^	3.4^ab^	3.6^a^	3.3	0.001
		(2.5–5.5)	(1.6–4.0)	(2.7–4.6)	(2.5–4.8)		
Indirect energy input	GJ ha^−1^ a^−1^	3.1^b^	4.7^c^	10.0^a^	10.8^a^	7.2	***
		(1.3–6.0)	(2.6–7.0)	(7.1–12.4)	(8.8–13.3)		
Seed	GJ ha^−1^ a^−1^	1.1^b^	0.4^a^	0.8^b^	0.4^a^	0.6	***
		(0.6–1.9)	(0–0.9)	(0.5–2.1)	(0–0.8)		
Plant protection products	GJ ha^−1^ a^−1^	0.1^a^	0^a^	1.4^c^	0.5^b^	0.4	***
		(0–0.5)	0	(0.8–2.1)	(0–1.1)		
Mineral fertilizer	GJ ha^−1^ a^−1^	0.1^a^	0^a^	6.3^c^	3.5^b^	2.4	***
		(0–0.9)	(0–0.4)	(4.2–9.0)	(0–8.4)		
Organic fertilizer	GJ ha^−1^ a^−1^	1.2^a^	3.6^b^	0.9^a^	5.5^c^	3.1	***
		(0.1–3.1)	(1.1–5.9)	(0–3.5)	(2.0–10.2)		
Machinery	GJ ha^−1^ a^−1^	0.6^ab^	0.6^ab^	0.6^a^	0.8^b^	0.7	0.018
		(0.4–0.9)	(0.3–1.1)	(0.5–0.9)	(0.4–1.3)		
Energy output	GJ ha^−1^ a^−1^	72.5^a^	120.0^b^	151.3^c^	185.4^d^	136.4	***
		(51.6–113.7)	(80.5–165.0)	(109.7–195.3)	(127.3–226.3)		
EUE		11.2^a^	16.5^b^	11.4^a^	12.9^a^	13.3	***
		(6.8–14.6)	(12.2–26.2)	(8.4–14.7)	(10.5–15.6)		
EUE without organic fertilizer		13.2^a^	33.2^c^	12.4^a^	22.9^b^	22.0	***
		(8.4–18.5)	(14.5–61.5)	(8.7–16.8)	(13.4–42.6)		

Units in parentheses represent minimum and maximum values, respectively. *p*—probability value was obtained using the F-test. Differences between farm types denoted by the same letter (a–c) were not significantly different at the 0.05 probability value calculated in post-hoc comparisons using the Tukey HSD test, ***p < 0.001.

The indirect energy inputs were significantly higher in CF (arable CF 10.0 GJ ha^−1^, dairy CF 10.8 GJ ha^−1^). These significant differences were caused by the use of mineral fertilizers and plant protection products that require a lot of energy in their production. Dairy farms had a significantly higher input of organic fertilizers in comparison to arable farms. In addition, the input on organic dairy farms was significantly lower than in CF because CF had higher stocking rates.

**Figure 2:**
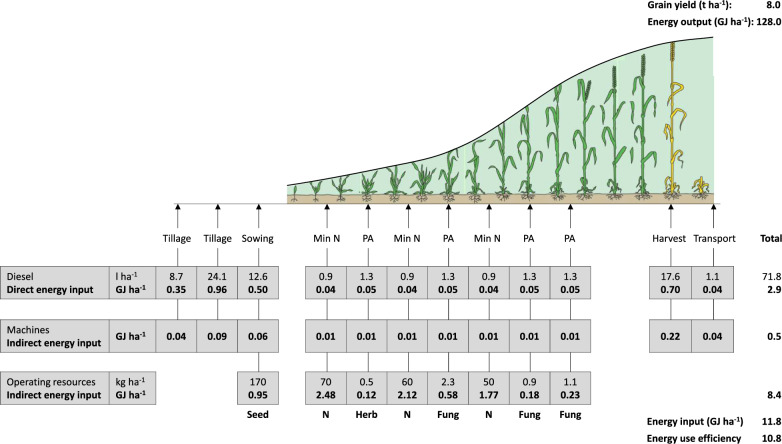
Energy input assigned to various farm operations for the production of winter wheat. Example of a conventional pilot farm. Explanations: Min-N: Mineral N application, PA: Pesticide application, Herb: Herbicide, Fung: Fungicide.

**Figure 3:**
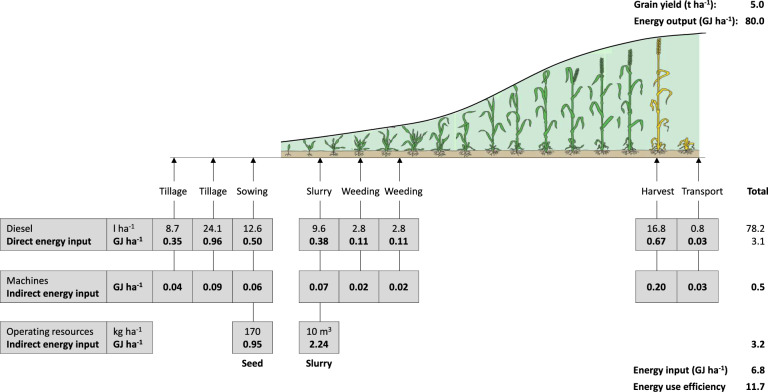
Energy input assigned to various farm operations for the production of winter wheat. Example of an organic pilot farm.

The highest indirect energy input came from mineral fertilizer (6.3 GJ ha^−1^) in arable CF and organic fertilizer (5.5 GJ ha^−1^) in dairy CF. Significant differences among the farming systems were found in the following indirect energy inputs: seed, plant protection products, mineral fertilizer, and organic fertilizer. There were no differences in energy input from the use of machinery. Nevertheless, there was a large variability between individual farms, partly also related to system differences, e.g. higher energy input for seeds on arable farms due to the lower proportion of grassland. Examples of energy inputs assigned to various farm operations for conventional and organic pilot farms are shown in Figs. 2 and 3.

### Energy output

Energy output depends on many factors, particularly the yield of the crop species grown, product quality (biomass energy content), biomass use (crop vs. straw and green mulch), and cropping structure. The substantial differences in these factors (Table 1) explain the extremely large differences in energy input. In all comparison pairs, the energy output was lower in OF (101.0 GJ ha^−1^) than in CF (171.8 GJ ha^−1^). Energy output in OF ranged from 51.6 to 165.0 GJ ha^−1^, in CF from 109.7 to 226.3 GJ ha^−1^ (Table 3).

Generally, energy output was lower in OF systems and on the arable farms. The energy outputs differed significantly among the farming systems. The lowest output was recorded in organic AF (72.5 GJ ha^−1^), followed by organic DF (120 GJ ha^−1^) and conventional AF (151.3 GJ ha^−1^). Conventional DF had the highest output (185.4 GJ ha^−1^). There was also extraordinarily large individual farm variability. Some OF exceeded the energy output of CF.

**Figure 4:**
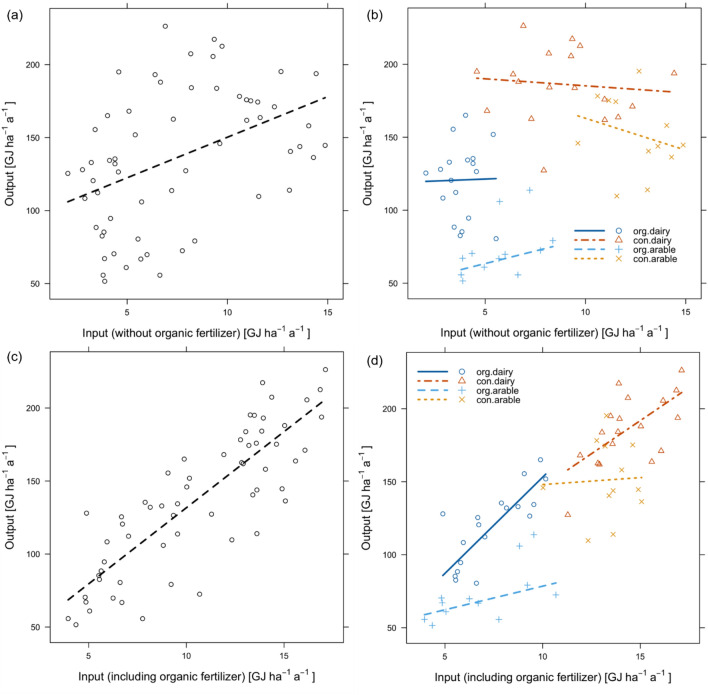
(**a**) Overall relationship between total energy input and energy output without organic fertilizer, (**b**) the relationship differentiated according to farming system, (**c**) overall relationship between energy input and energy output including organic fertilizer and (**d**) the relationship differentiated according to farming system.

Figure 4 shows the relationship between total energy input and energy output without organic fertilizer. The correlation between the parameters differed according to farming system. The relationship between energy input and energy output changed when organic fertilizer was included.

### Energy-use efficiency

The EUE of conventional farms was higher than on organic farms in only 19% of the comparison pairs (Fig. 1). The mean EUE (with organic fertilizers taken into account) in organic farming was 14.4 and in conventional farming 12.3 (Table 3). EUE of conventional farms ranged from 8.4 to 15.6. 10% of organic farms had an EUE comparable with conventional farms. In 71% of the farm pairs, the organic farms were more energy efficient than the conventional farms. When organic fertilizers were not taken into account, the EUE was higher, in OF 25.2 (from 8.4 to 61.5), and in CF 18.7 (from 8.7 to 42.6).

Generally, increasing energy input increased energy output in all systems when organic fertilizers were taken into account (Fig. 4). To achieve high energy output per unit area, sufficient energy input (sufficiently high production intensity) must be realized, this applies equally to OF and CF. To intensify sustainably, higher energy inputs in OF are required to increase energy output and to reduce the yield gap. OF systems are mainly low input systems (< 10 GJ ha^−1^), whereas CF farms are high-input systems (> 10 GJ ha^−1^).

**Figure 5:**
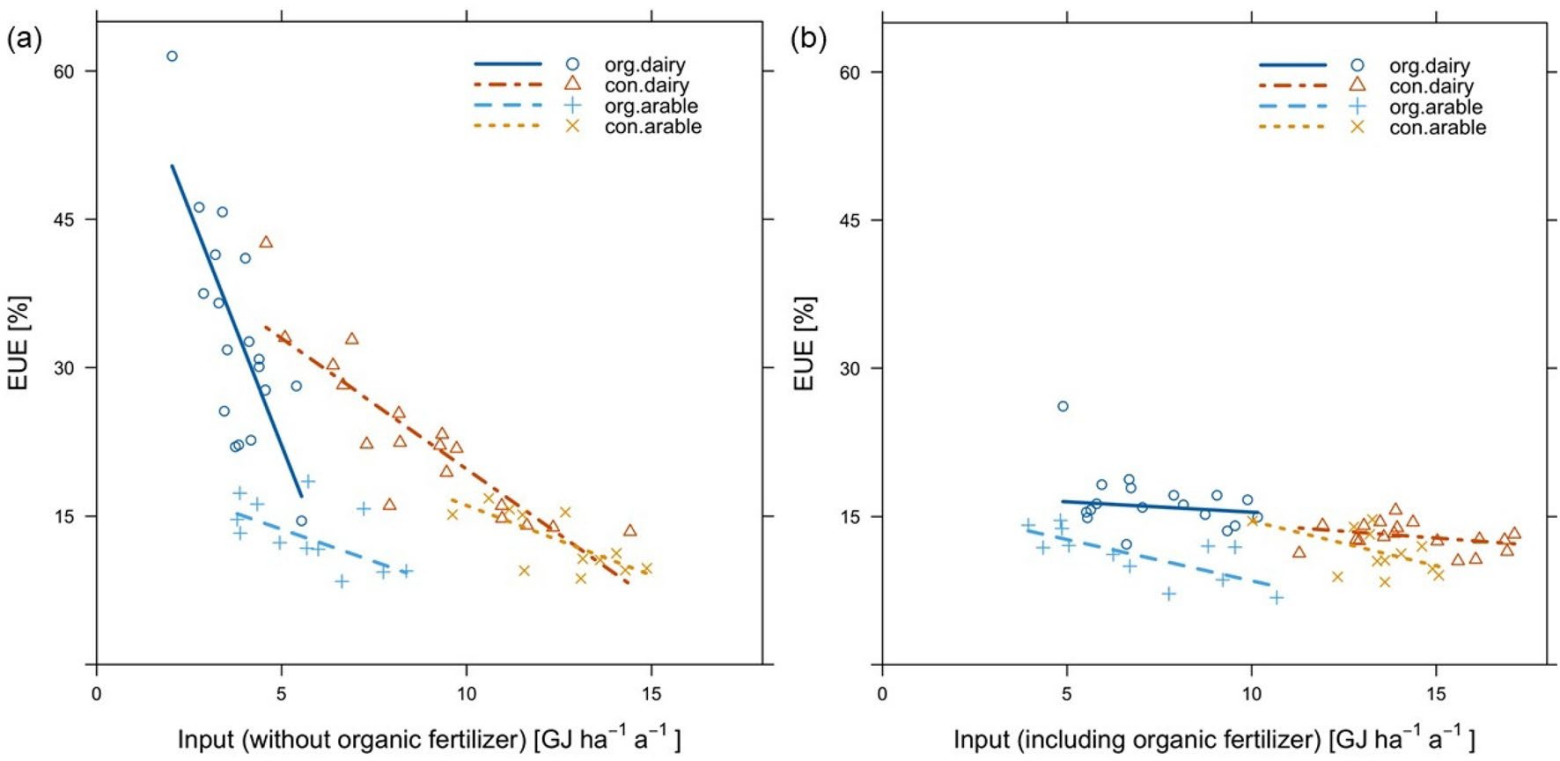
Farming system-specific relationship between energy input and EUE (**a**) without organic fertilizers and (**b**) including organic fertilizer.

The EUE was significantly higher on organic dairy farms (16.5) compared to the other farms (arable OF 11.2, arable CF 11.4, dairy CF 12.9). The inclusion or exclusion of organic fertilizer also affected EUE (Fig. 5).

## Discussion

Energy balances (energy input, energy output and EUE) were analysed in 30 pairs of organic and conventional arable and dairy farms in Germany. The energy fluxes of the systems were different because of systemic differences between the farming systems. The farming systems are complex, with many factors influencing energy efficiency that need to be taken into account in energy balances. Our results from 60 pilot farms in Germany, evaluated using a detailed, consistent energy balancing method, show considerable heterogeneity and differences, not only between farming systems and farm types, but also between farms (e.g. depending on grassland proportion, crop rotation, animal stocking rate, and fertilizer input). This variability suggests that the energy balance could be improved on some farms, and the methods used on highly efficient farms could show how this can be achieved.

A methodological problem with farm-level studies (on-farm research) is that they cannot be replicated (like plot trials) due to the specific conditions on the farms. This limits the possibilities for extrapolation of results to other farms. On the other hand, these studies of real farming systems have many advantages in comparison to field experiments. In field trials, usually, only a few intensities (often not relevant to farming in practice) are evaluated. On the pilot farms, there is a continuum from low to high energy input systems. The real farms show actual conditions in farming practice, the actual structures, energy inputs, and yields. Our results are a "snapshot" valid at the time of the analysis (2009–2011). The energy balances will continue to develop. Due to climate change mitigation and the need to lessen the (political) dependence on fossil energy, the use of fossil energy will probably be massively reduced in the future.

Within the systems, there were variations between individual farms depending on intensification level and the energy inputs (diesel, machinery, seed), partly also system-related differences, e.g. higher energy input for seed on arable farms because of the lower proportion of grassland. In organic AF, relatively high diesel consumption was due to high tillage intensity (ploughing) and intensive mechanical weed control. Tillage affects the total energy input, e.g. Monteleone et al. ^42^ recorded a 10% reduction in the total energy input due to no-tillage management and direct sowing. In organic DF, lower diesel use is explained by the high proportion of meadows and pasture (energy-extensive systems), as also suggested by Gaudino et al.^27^.

According to Pimentel et al.^43^, the energy inputs on organic arable and dairy farms were about 30% lower than in conventional systems. However, the results are from the Rodale Institute Farming systems trial. Our results from real farms suggested 50% lower inputs. Further, the dominant high energy input in conventional agricultural systems is mineral nitrogen fertilizer, on organic arable farms the direct input of fuel. These results are in accordance with Helander and Delin^39^. Fertilizer application rates were often higher than recommended, as described by Han et al.^44^ in a meta-analysis. However, this problem was not observed on the pilot farms. The nitrogen surpluses of the pilot farms, described in Chmelíková et al.^45^, were much lower than the national average, and the mineral N inputs were well adapted to the farm structure, the level of organic fertilization and the yields (e.g. significantly lower mineral N use on the conventional dairy farms than on arable farms). However, in regions with a high livestock density (more than 2 LU/ha) and overfertilization, a lower energy efficiency than on the pilot farms is to be expected^46^.

As suggested by Amenumey and Capel^34^, alternative sources of fertilizers exist and, in general, more efficient use of fertilizers could decrease the use of fossil energy. In this context, the problem of accounting for organic fertilisers is described in Godinot et al.^47^. The status of manure varies from a waste product (in regions with massive manure surpluses) to a highly valuable resource (in intensive cropping regions with low soil organic matter). All energy balances were calculated with and without energetic evaluation of the organic fertilizers to enable the comparison with other studies (organic fertilizers are usually not taken into account). The production of organic fertilizer costs fossil energy and the use of organic fertilizer has effects on crop yields, so it must be taken into account (e.g. as a substitution value, such as in this study). If organic fertilizers were not taken into account in energy balancing, the yield and energy output would increase without energy input. If complete farming systems (crop production and dairy farming) are analysed, however, care must be taken in this context to avoid double counting of the energy input^41^.

Further, the energy input was affected by the crop rotations. For example, Hülsbergen and Kalk^48^ and Entz et al.^49^ found that the inclusion of legumes in crop rotations had an effect on energy input, energy output, and EUE. The cultivation of legumes resulted in higher grain yields, helped reduce mineral nitrogen inputs (i.e., reduced energy inputs), and improved energy efficiency. On the other hand, the cultivation of cover crops increased energy inputs due to the use of seed and additional field operations. However, this energy cost can be compensated by benefits for soil and subsequent crops, as suggested by Abdalla et al.^50^.

Energy output was lower in OF than in CF, and lower on arable farms than on dairy farms. Many authors have analysed the yield gap between organic and conventional farms^36,51–54^. Various strategies could be used to close this yield gap and reduce the difference in energy output between organic and conventional systems. Appropriate plant breeding may further improve cereal yields in organic farming. According to our results, an increase in energy output requires a corresponding increase in energy input. However, when energy input increases, energy-use efficiency often decreases. It is extremely difficult to optimize energy output and energy efficiency at the same time. Therefore, a decision usually has to be made as to whether energy output or energy-use efficiency has priority^55^. Since land is a limiting (scarce and expensive) factor, there is a strong case for increasing energy output, even in organic farming.

Generally, a high EUE was recorded in dairy farming systems. Organic dairy farming systems were more efficient than organic arable farming systems. Similar results and the many advantages of linking arable and dairy farming systems were described by Gaudino et al.^27^ and Wilkins^56^. Organic farming is mainly dependent on the low energy inputs typical for extensive dairy farming systems. The EUE of organic dairy farming systems is affected by the presence of pasture because very little energy is used in grazing. Clover grass in particular can increase energy output and energy efficiency because high forage yields are possible without nitrogen fertilisation. Further, double cropping increased energy output with low input. All in all, many authors^27,57,58^ reported higher EUE in OF for the whole farming system, crop rotation or for individual crops, e.g. winter wheat^21,32,59,60^.

EUE can be increased by reducing fossil energy inputs (e.g. using renewable energy, optimized management processes, use of energy-efficient technologies) and increasing yield and energy retention (e.g. efficient crop varieties, optimization of crop rotations). A reduction in the use of fossil energy is urgently required to mitigate climate change and to reduce political dependence on energy imports into the EU^61,62^. Agriculture must also make a contribution to reducing fossil energy use, raising the question as to whether the high energy inputs for mineral nitrogen production can be maintained to the extent practiced to date.

EUE has increased in Europe over the last few years, with a corresponding reduction in CO_2_ emissions^63^. Higher EUE can be mainly attributed to improved machinery and farming practices, an increase in the use of renewable energy sources, recycling of agricultural residues, and cooperation between farms. A comparison of the energy-flux efficiency of different farming systems might give a deeper insight into sustainable resource use and environmental performance.

The results show energy balances of the entire crop production system of the farms, not of the individual crops. Not every farm cultivated every crop species and this would limit the evaluation of the data set. The entire crop production system was analysed, with crop rotations and the proportion of grassland specific to each location and typical for each system (organic and conventional, arable and dairy farming system). The main factors influencing the results were the differences in the principles and methods of organic and conventional farming, namely the farm structures and management intensity, especially fertilization intensity. The energy inputs in OF and CF differed due to the different N inputs, tillage intensity and pesticide use, and were dependent on soil conditions, crop development, and disease pressure from year to year.

## Methods

### Study area

**Figure 6:**
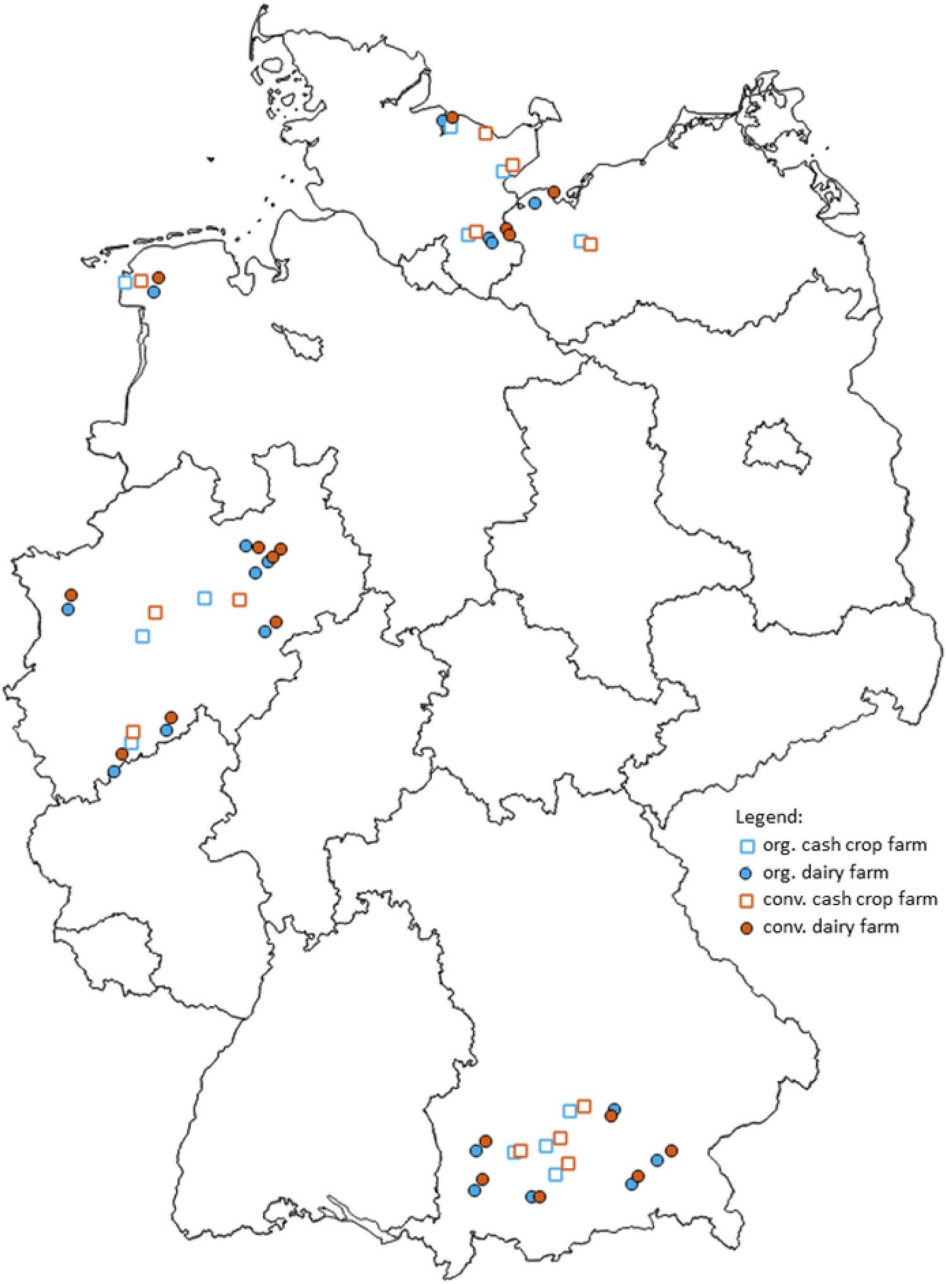
Location of 30 farm pairs (one organic and one conventional farm) in Germany analysed in this study. In total 60 farms, 18 dairy farm pairs and 12 arable farm pairs^45^. The figure ( © GeoBasis-DE/BKG (2023); modified with own data) was generated with QGIS^67^.

Data were collected from 30 organic and 30 conventional farms from the network of pilot farms (Fig. 6) in three German regions (south, west, and north), starting in 2009. The data came from the project “Ecological Sustainability of Agricultural Systems—Analyses in a Network of Pilot Farms”. The project aim was to analyse the environmental sustainability and resource-use efficiency of the farming systems. One focus of the project was the analysis of energy balances. In this study, the focus is on the energy balance of plant production (cash crop production, fodder production) on arable land and grassland for the period 2009–2011. The network consists of pairs of farms that are located near each other in the same pedoclimatic area. Organic farms had to have been under organic management for at least seven years.

In each region, two farm types (arable (AF) and dairy farm (DF)) were present. The crop production systems (e.g. proportion of arable land and grassland, fertiliser use, production methods) of these farm systems differ significantly (Table 1).

Farm elevation in the southern region of Alpine Foreland ranged from 444 to 776 m, in the western region with a maritime climate from 21 to 421 m, and in the northern coastal region from − 4 to 52 m. Farms with differing site and climate conditions were chosen in order to represent a wide range of management conditions.

Besides regional aspects, selection criteria for the pilot farms were that the farmer worked on the farm full-time, had comprehensive and precise data documentation (field records, livestock husbandry data), made data available and was willing to actively participate in the project. Farm size had to be equal to or larger than the average size of the farms in the region. In this study, 12 organic and 12 conventional farms are arable farms, and 18 organic and 18 conventional farms are mixed dairy farms, combining dairy and arable farming. The energy balances are used to analyse crop production in order to enable a system comparison. Animal husbandry is not included in the energy analyses.

There were only small differences between organic and conventional farms regarding other characteristics such as farm size or soil quality (Table 1).

### System modelling and energy balancing

Energy flux calculations were computed for plant production systems for the initial phase of the project period 2009–2011 before the energy use and resource efficiency of farms were optimized. The complete plant production process on each pilot farm was assessed using detailed interviews. Generally, the methodological approach is described in detail in^48,64,65^.

**Figure 7:**
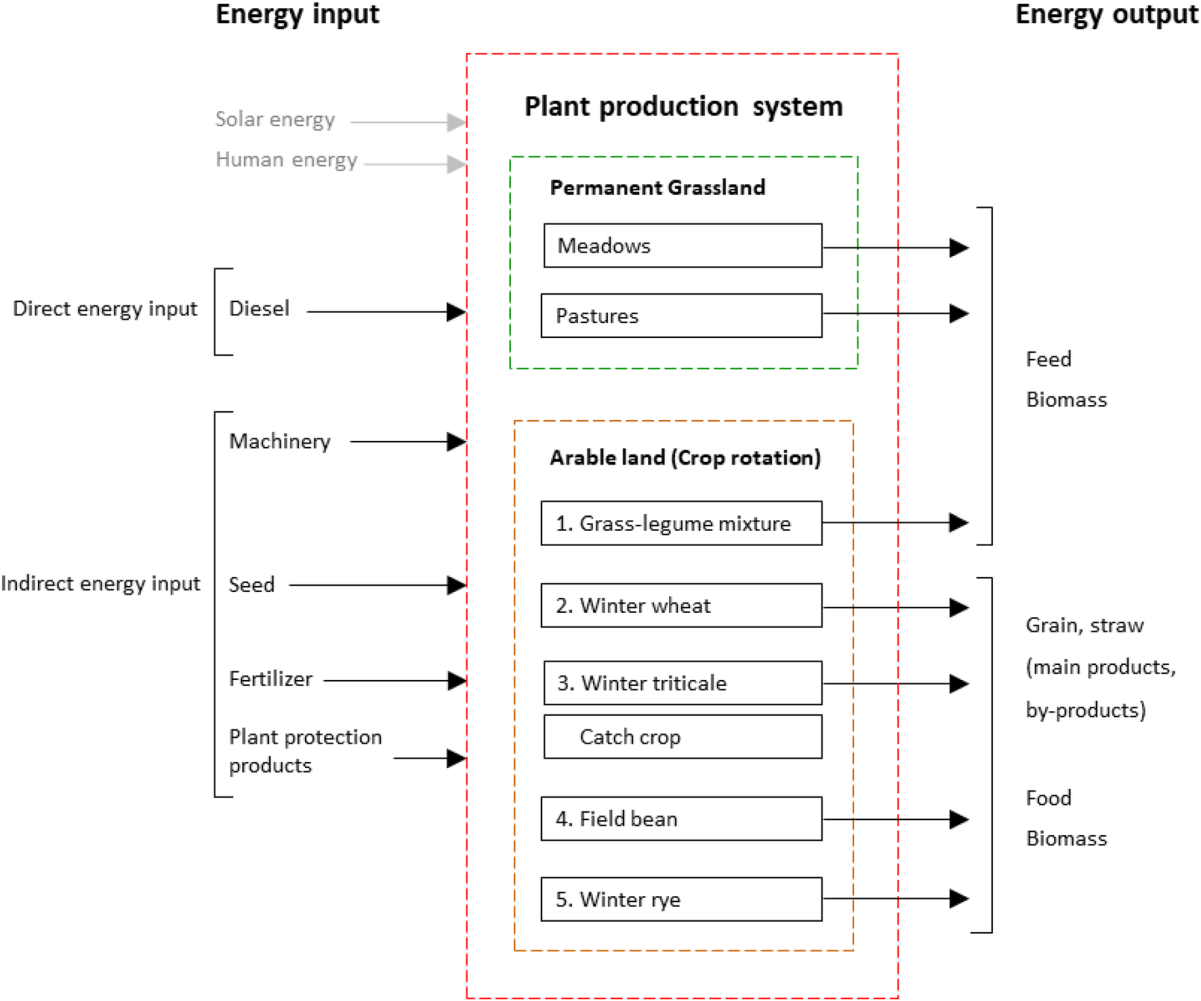
Diagram showing energy inputs and outputs of plant production systems. Solar and human energy is not included in the analysis.

All relevant data for all fields and all crops on arable land as well as for permanent grassland, i.e. all relevant data for the production processes (tillage, fertilization, crop protection, etc.), were collected on all farms. These data were used for the modelling of energy fluxes with the model REPRO^66^. REPRO is a model used to analyse material and energy flows in crop production. It has an integrated method for calculating energy balance based on available farm data, attempting to trace all fossil energy inputs into a plant production system based on physical material flows (Fig. 7). In the model, we use process analysis for the calculation of energy fluxes. The modelling approach used in this study is described in detail in Hülsbergen et al.^48^, Küstermann et al.^59^, and Deike et al.^32^. The analysis of the plant production systems was carried out at the field level. Therefore, only the crop production subsystem and not the animal production subsystem was taken into account. In Fig. 7, the system boundary with inputs and outputs is shown. The parameters in this study were energy input, energy output, and EUE. The energetic parameters and their calculations are summarized in Table 4.

**Table 4:** Definition of energetic parameters.

Energetic parameter	Definition	Unit
Direct energy input (E_d_)	Diesel input	GJ ha^−1^ a^−1^
Indirect energy input (E_i_)	Seed + mineral and organic fertilizers + pesticides + machines	GJ ha^−1^ a^−1^
Energy input (E)	E = E_d_ + E_i_	GJ ha^−1^ a^−1^
Energy output (EO)	Energy in the harvested biomass (main product + by-product) − energy in seed	GJ ha-^−1^ a^−1^
Energy-use efficiency (EUE)	EUE = EO/E	–

The energy inputs included both direct and indirect energy inputs. The direct energy is the energy used on the farm (fuel, electricity). The indirect energy is energy used outside of the farm for the manufacture of fertilizers, plant protection products, machinery, etc.^48^. For organic fertilizers, the substitution value is used. Based on the nutrient content (N, P, K) of the organic fertilizers and their effectiveness in comparison to mineral fertilizers (mineral fertilizer equivalents), the primary energy requirement that would be necessary to produce the same amounts of mineral fertilizer is determined. The energy input for producing machines and equipment is determined and then allocated to one working hour or one hectare. The normative useful/service life is assumed here (analogous to depreciation in economics). The packaging and transport of these materials are also included in indirect energy. To include the input of energy associated with the manufacture, packaging, and transportation of production means in terms of primary energy input, energy equivalents were used^48^ and are shown in the supplemental materials. Energy equivalents were used to account for both direct and indirect components, according to the nature of the input. These equivalents are widely-used values representing mean German conditions at the time and for the region (e.g. the average energy input for the production of mineral nitrogen fertilizer) and hence are appropriate for this study. We did not include environmental inputs (e.g. solar energy) or human labour energy. As the differing crop management systems are the focus of our evaluation of energy flows and energy-use efficiency, we use the field border as the system boundary of our energy analysis. Consequently, energy in farm buildings, crop grain drying and storage, and further components of the feed and food chains were not included in our analysis. Such energy consumption would occur regardless of which crop management technique was used.

The energy output was calculated based on DM yields and the gross energy content (calorific values) of products. The harvested products (e.g. wheat grain, potatoes, sugar beets, corn silage, etc.) were included in our energetic analysis. However, how these products were used was not included. The unharvested biomass (e.g. straw, leaves, residues, and green manure) is not considered in this study. Calorific values were derived from the product quality (the content of protein, fat, fibre, and N-free extracts), see Hülsbergen^64^. The mean calorific values used in this study for crops are shown in the supplemental materials.

### Data analysis

The percentage differences in energy input, energy output, and energy-use efficiency of organic and conventional farming systems were evaluated. A difference was defined as relevant if the organic and conventional values differed by + / − 10%. Loess smoothing was used to analyse the relationships between energy input and energy output. Boxplots for these percentage differences and the relationships were created using R 3.3.3 (R Core Team 2017).

One-way ANOVA was used to evaluate the effects of farming systems and farming types. After obtaining significant results, multiple comparisons using Tukey’s HSD test were applied to identify significant differences among the four different variants (OF-CC; OF-DF; CF-CC; CF-DF). The analysis was conducted with all the values of energy input, energy output and energy-use efficiency for the farming systems. The analysis was performed using STATISTICA 12 software.

### Use of plant material and animals

No specific collection of plant material or analysis involving animals was conducted. Data on crop yield and animal stocking were provided by farmers derived their standard farming practices. Thus, all relevant institutional, national, and international guidelines and legislation were met and no special permissions and/or licences were needed.

## Conclusion

Our study compared EUE of organic and conventional farming systems. Generally, OF had lower energy inputs and energy outputs in comparison to CF. Lower input conserves resources (e.g. fossil energy for fertilizer production) and protects the environment (e.g. less greenhouse gas emissions). The differences between OF and CF resulted primarily from the specific type of internal processes (N_2_ fixation by legumes, humus management, development of soil fertility, nutrient cycles etc.) of OF. In CF, mineral nitrogen fertilizer was often the most important energy input. There is no clear distinction between organic and conventional crop production. Instead, there are many variations within the systems, depending on site conditions, farm structures, process design and management. This is also reflected in the energy input, which increases continuously from extensive organic to intensive conventional farms. In future, more attention should therefore be paid to the variability within the systems. Our results show that a further expansion of organic farming area (in Germany to 30% by 2030, in the EU to 25% by 2030) would significantly reduce energy input in agriculture. This would also be associated with lower GHG emissions. However, yields and energy use would also decrease, with the decrease larger for organic arable farms than for dairy farms. To ensure intensification is sustainable, higher energy inputs in OF are required to increase energy output and to reduce yield gap.

The energy balances will continue to develop. Climate change mitigation and decreasing the EU´s dependence on fossil energy will probably massively reduce the use of fossil energy in the future.

### Supplementary Information

Supplementary Tables.

## Data Availability

The datasets generated and/or analysed during the current study are not publicly available due privacy reasons but are available from the corresponding author on reasonable request.
