# Supplementary material for: Energy-use efficiency of organic and conventional plant production systems in Germany
Source: Sci Rep. 2024 Jan 20;14:1806. doi: 10.1038/s41598-024-51768-3 (PMC10799894; doi:10.1038/s41598-024-51768-3)
Supplement: Supplementary file 1 — Supplementary Tables. [file 41598_2024_51768_MOESM1_ESM.pdf]

Table 1: Definition of energetic parameters

Energetic parameter	Definition	Unit
Direct energy input (E_d)	Input of diesel	GJ ha^{-1} yr^{-1}
Indirect energy input (E_i)	Seed + mineral and organic fertilizers + pesticides + machines	GJ ha^{-1} yr^{-1}
Energy input (E)	$E = E_d + E_i$	GJ ha^{-1} yr^{-1}
Energy output (EO)	Energy in the harvested biomass (main product + by-product) – energy in the seed	GJ ha^{-1} yr^{-1}
Energy use efficiency (EUE)	EUE = EO / E	--

Energy-use efficiency is defined as energy output in relation to energy input. A definition of the energetic parameters and their calculation is given in Table 1.

The energetic analysis was done with a process analysis. This is a more mechanistic technique, attempting to trace all fossil energy inputs into an agricultural system based on physical material flows, and is suitable for calculating energy balance, analyzing energy-use efficiency, and comparing and improving farming systems (Hülsbergen et al., 2001; Küstermann et al., 2008; Alluvine et al., 2011; Rossner et al., 2014; Jankowski et al., 2015). We analyzed the plant production of farming systems at field level. Therefore, for organic mixed farming system, only the crop production subsystem was included but not the animal production subsystem.

The system inputs and outputs and system boundary are shown in Figure xy. The energy inputs considered both direct energy and indirect energy. Direct energy is the energy used on the farm (fuel, electricity), and indirect energy is energy used outside of the farm for the manufacture of fertilizers, plant protection products, machinery, etc. (Hülsbergen et al., 2001). The packaging and transportation of these materials are also parts of the indirect energy.

To include the input of energy associated with the manufacture, packaging, and transportation of production means in terms of primary energy input, energy equivalents were used (Hülsbergen et al., 2001).

Table 2: Energy equivalents used in this study. The energy inputs associated with the manufacture, packaging, and transportation of production means are included in the energy equivalents.

**Energy equivalent**	**MJ kg^{-1} (kg DM^{-1} for seeds)**	**References**
Machinery	108	Kalk and Hülsbergen (1996); Rossner et al. (2014)
Diesel	39.6	Reinhardt (1993); Rossner et al. (2014)
Mineral N	35.3	Appl (1997); Hülsbergen et al. (2001); Rossner et al. (2014)
P fertilizer	36.2	Kaltschmitt and Reinhardt (1997); Kukk et al. (2011)
Herbicides	288	Green (1987); Deike et al. (2008); Alluvine et al. (2011); Rossner et al. (2014)
Insecticides	237	Green (1987); Deike et al. (2008); Alluvine et al. (2011); Rossner et al. (2014)
Fungicides	196	Green (1987); Deike et al. (2008); Alluvine et al. (2011); Rossner et al. (2014)
Wheat seed	6.5	Hülsbergen (2003)
Potato seed	5.7	Hülsbergen (2003)
Maize seed	17.0	Hülsbergen (2003)
Grass-clover-alfalfa seed	18.0	Hülsbergen (2003)
Sunflower seed	12.0	Hülsbergen (2003)
Rye seed	6.5	Hülsbergen (2003)
Mustard seed	12.0	Hülsbergen (2003)

These equivalents are the widely-used values for representing the mean German conditions relevant and modern technologies, and hence are appropriate for this study (Lin et al. 2016).

Energy equivalents were used to express the input of energy associated with the manufacture of production means in terms of primary energy input. There is an enormous variation in energy equivalents reported in the literature. This is the result of differences in the methods of calculation and in the spatial and temporal system boundaries. Energy equivalents are not fixed once and for all. They must be adapted to local conditions (e.g. transport distances) and to changes in the manufacture of production means. Special emphasis must be put on the energy equivalents of fertilizers, because the rate of fertilizer application has a particularly strong effect on the energy input (Hülsbergen et al. 2002).

Table 3: Mean calorific values used in this study. They were derived from the product quality (the content of protein, fat, fiber, and N-free extracts).

**Mean calorific values**	**MJ kg DM^{-1}**	**References**
Potato tubers	17.2	Hülsbergen (2003)
Wheat grain	18.6	Hülsbergen (2003)
Forage maize	19.0	Hülsbergen (2003)
Rye grain	18.3	Hülsbergen (2003)
Sunflower grain	26.8	Hülsbergen (2003)

[a] Calculated according to Schiemann (1981): gross energy (MJ) = crude protein (kg) × 23.9 (MJ kg^{-1}) + crude fat (kg) × 39.8 (MJ kg^{-1}) + crude fiber (kg) × 20.1 (MJ kg^{-1}) + N free extracts (kg) × 17.5 (MJ kg^{-1}). [b] DM, dry matter, FM, fresh matter

Energy output was calculated on the basis of DM yields and the gross energy content (calorific values) of products. We analyzed the harvested products (e.g. wheat, GCA) independent of the use of these products. However, the non-harvested biomass (e.g. straw, leaves, residues, and green manure) are not accounted in this study (see Table 1). Calorific values were derived from the product quality (the content of protein, fat, fiber, and N-free extracts, see Hülsbergen et al. (2001)).
